# Influence of Surface Preparation on the Microstructure and Mechanical Properties of Cold-Sprayed Nickel Coatings on Al 7075 Alloy

**DOI:** 10.3390/ma16217002

**Published:** 2023-11-01

**Authors:** Wojciech Żórawski, Anna Góral, Medard Makrenek, Lidia Lityńska-Dobrzyńska, Paweł Czaja

**Affiliations:** 1 Faculty of Mechatronics and Mechanical Engineering, Kielce University of Technology, Tysiąclecia Państwa Polskiego 7, 25-314 Kielce, Poland; 2 Institute of Metallurgy and Materials Science, Polish Academy of Sciences, Reymonta 25, 30-059 Krakow, Polandl.litynska@imim.pl (L.L.-D.); p.czaja@imim.pl (P.C.); 3 Faculty of Management and Computer Modelling, Kielce University of Technology, Tysiąclecia Państwa Polskiego 7, 25-314 Kielce, Poland

**Keywords:** cold spraying, nickel, microstructure, mechanical properties

## Abstract

This work presents the effect of surface roughness (Al 7075) on the microstructure and mechanical properties of cold-sprayed nickel coatings. Coating analysis included substrate surfaces and coating geometry, microstructure characterization, microhardness, nanohardness, elastic modulus, and adhesion. The results show that the surface preparation had a significant effect on coating adhesion and microstructure. The coating deposited at the highest gas temperature revealed a dense microstructure, showing very good adhesion of the impacting powder particles to the substrate and good bonding between deposited layers. The Ni grains with different shapes (elongated, equiaxed) and sizes of a few dozen to several hundred nanometres were present in the splats. An increase in temperature caused significant growth in coating thickness as a result of the powder grains’ higher velocity. Moreover, higher gas temperature resulted in the enhancement of micro- and nanohardness, elastic modulus, and adhesion. The adhesive bond strength of Ni coatings in the tested temperature ranges from 500 °C to 800 °C increased with the increase in the surface roughness of the substrate. For the Al 7075 coarse grit-blasted (CG) substrate with the highest roughness, the adhesion reached the highest value of 44.6 MPa when the working gas was at a temperature of 800 °C. There were no distinct dependencies of surface roughness and thickness on the mechanical properties of the cold-sprayed nickel coating.

## 1. Introduction

Recently, the cold spray (CS) technique has been widely investigated as it allows high-quality coatings to be obtained with enhanced properties. CS has the advantage of minimizing the detrimental effects of high-temperature processing, such as oxidation, porosity, and phase transformation, compared to other thermal spray technologies, which involve either partial or complete melting of particles during deposition [1,2,3,4,5]. As a low- temperature process, CS allows the original powder chemistry and phases in the coating to be retained. During cold spraying, localized severe plastic deformation of particles takes place at a high strain rate, resulting in both the strain hardening and heating up of materials. The formation of bonds between a particle and the substrate or the previously deposited layer requires a critical impact velocity of particles, which is a function of the spray material and its plastic deformation characteristics. Furthermore, successful particle−substrate and particle−particle bonding requires a high level of plastic deformation and adiabatic shear instability [6,7,8,9]. The density and impermeability of the cold- sprayed coatings depend strongly on the powder characteristics and spray conditions and parameters. Materials with good deformability and high density, e.g., Cu and Zn [3,8,9], are susceptible to cold spraying of dense coatings, whereas, for materials of relatively high strength, such as Ni [10,11], manufacturing of dense coatings requires the application of higher preheating temperature of the process gas. Dense coatings of nickel and its alloys reveal good corrosion resistance and, therefore, have a high potential for employment as corrosion barrier layers [11,12]. Moreover, nickel is believed to reduce fretting corrosion as a result of the so-called ‘‘glaze’’ oxide layer formed at high temperatures, which is reported to reduce friction and wear in nickel-based alloys [13,14]. Additionally, high-performance electrodes used in silicon solar cells may be fabricated through cold spraying of nickel and copper particles [15]. Hence, obtaining dense and oxide-free Ni coatings requires great effort. Additionally, appropriate substrate surface preparation, resulting in longer manufacturing times of the coating, should be taken into account. The most common way is sand-blasting (grit-blasting) with different kinds and sizes of grit. Unambiguous determining of the applied sand-blasting influence on changes in the microstructure and properties of coatings are still not known. A few papers [2,14,15,16,17,18,19] concerning this problem presented opposing results depending on the used powder feedstocks and substrate materials. As can be expected, blasting by coarse grit leads to greater surface roughness than by finer sand because each individual grit impact causes more plastic deformation of the substrate surface.

As reported by Richer [16], a larger substrate surface roughness results in a greater substrate surface area, and therefore, more particles creating the coating come into direct contact with the substrate during the spraying of the initial layer. This causes a higher deposition efficiency for the initial layer of sprayed material, which is not observed for the next layers. Moreover, it does not influence the coating porosity content. The effect of using different grit sizes for the substrate preparation is limited to small changes in the deposition efficiency of only the first few layers of the deposited material. Bruera et al. [17] achieved increased adhesion of Cu coatings on medium grit-blasted AISI 304 soft stainless steel. A different effect was obtained by Nastic et al. [18], where the Al coating on grit-blasted low-carbon steel had reduced adhesion. Cu coatings sprayed on smooth 316 stainless steel also had better mechanical and physical properties [19]. Marrocco et al. [20] showed that Ti coatings deposited on Ti_6_Al_4_V substrates ground with SiC paper have higher bond strengths than those on grit-blasted Al_2_O_3_ substrates. This is caused by the hardening of the surface during grit-blasting, which limits the deformation of the substrate by impacting Ti particles and thus hinders the formation of a primary bond. Similar results were presented by Husain et al. [21] for Cu coatings deposited onto an Al substrate. They showed that adhesion of the coating and a grit-blasted substrate was significantly reduced by grit embedment into the aluminum surface during blasting. In turn, Jingwei et al. [22] found micropores and defects in the interface of the Al-Si coating, and a grit-blasted mild steel surface resulted in observed lower bond strengths. In addition, Yin et al. [23] showed that a Ni coating deposited on a polished and ground Al substrate provides higher coating mass and coating−substrate bonding strength than one formed on a grit-blasted substrate. Contrary results concerning Cu and Ti coatings cold sprayed on mild steel substrates are reported by Sakaki et al. [24], showing that the deposition efficiency of metallic powders increases slightly with a greater roughening of the substrate surface (going from polished to grit-blasted). Furthermore, Kumar et al. [25] showed that bond strength values for grit-blasted aluminum or copper substrates and copper coatings are higher than the smooth cases due to the enhanced mechanical interlocking, which plays an important role in the bonding mechanism. As described above, many efforts have been devoted to studying the effect of substrate surface conditions on the coating−substrate bonding and deposition efficiency during the past years. It can be ascertained that plastic deformation significantly depends on the material properties of the feedstock and substrate. However, convincing evidence to unambiguously explain the effect of the surface substrate preparation on coating quality is still lacking. The aim of this study is to investigate the influence of different Al 7075 surface pretreatments on the microstructure and mechanical properties of cold-sprayed nickel coatings. Despite many experiments, there are limited investigations into the influence of grit-blasting on the mechanical properties of cold-sprayed Ni coatings. Moreover, grit-blasting is an optional treatment and can introduce unfavorable pollution at the interface in the form of small corundum grains. This process is applied in most experiments independently of powder−substrate relationships [26]. As a result, after cold spraying, the interface is contaminated, and coating adhesion is reduced. There is no recommendation concerning any pretreatment process of the substrate surface before cold spraying [27,28].

## 2. Materials and Methods

Nickel coatings were sprayed using an Impact Innovations 5/8 cold spray system equipped with a Fanuc M-20iA robot. Nitrogen was applied as the main gas with a working pressure of 3.0 MPa. The preheating temperature of nitrogen was 500, 600, 700 and 800 °C.

The distance between the nozzle exits and the sample surface was 60 mm. The traverse speed of the gun was 400 mm/s with a step size of 2 mm between 10 passes. The total range of cold spraying one sample included four layers to ensure sufficiently thick coatings. Ni coatings were deposited on Al 7075-T6 samples with the dimensions of 30 × 400 × 5 mm. The surface of the samples was divided into three parts along the length and prepared in three different ways. The first part was left in the delivery condition and marked NG (no grit-blasting). The surface of the middle part was grit-blasted by medium corundum grit with the size of 559 µm and designated as MG (medium grit-blasting). The last portion of the surface was blasted with coarse corundum grit with the size of 1600 µm and designated as CG (coarse grit-blasting). To conduct the experiment, 12 samples of nickel coatings were sprayed. Nickel powder used in the cold spraying process was Metallisation P836 (Ni > 99.5) with a grain size range of 11–45 µm. For comparison, a sample of solid nickel with the dimensions of 10 × 20 × 10 mm was cut off from the Ni electrode (marked Ni) by a wire-cut electrical discharge machine.

The microstructure of the Ni powder and cold-sprayed coatings was analyzed with the following microscopes: SEM FEI Nova^TM^ NanoSEM 200 (FEI Company, Hillsboro, OR, USA), E-SEM FEI XL 30 (FEI Company, Hillsboro, OR, USA), as well as TEM FEI TECNAI G2 (FEI Company, Hillsboro, OR, USA). For cross-section observations, both powders and coatings were embedded in a resin and polished using diamond suspensions of gradations 3 µm, 1 µm, and 0.25 µm. The Focused Ion Beam (FIB) technique using an FEI QUANTA 3D Dual Beam (FEI Company, Hillsboro, OR, USA), was employed to prepare the cross-sectional TEM specimens from coatings. They were cut out from the region near the surface coating and other regions covering both parts of the coating and Al alloy substrate. The phase composition was studied using D8 Discover Bruker (as sprayed deposits) and X’Pert Philips PW 1710 (powder feedstock) (PANalytical, Almelo, The Netherlands), diffractometers with Co-Kα radiation of wavelength λ = 1.78897 Å. The Vickers hardness HV0.3 was measured with a Matuzawa MMT-X3A microhardness tester (Matsuzawa Co., Ltd., Akita, Japan). The mean value of ten readings was taken for each coating. The micromechanical testing of coatings was carried out using the nano-indentation technique (Nanovea) with a Berkovich indenter (Olivier and Pharr methodology). Forty-nine readings were taken for each coating. The geometry of the sample surface before cold spraying and “as sprayed” coatings was analyzed with a Talysurf CCI-Lite noncontact 3D profiler [22]. Adhesion tests were performed using a Positest AT-A device (DeFelsko, NY, USA) with an automatically controlled hydraulic pump. An aluminum dolly was covered with glue and pressed onto the coating with 0.3 MPa. After 24 h at room temperature, the samples were subjected to pull-of tests. The test starts by attaching the actuator to the adhered dolly and initiating the measurement. When the detachment occurs, the maximum pressure value is shown on the instrument screen, which represents the adhesive strength of the coating. Two-component Loctite LT9466 resin was applied to glue the samples. In order to avoid the influence of the coating thickness on the adhesion test results, the coating was removed around the dolly by milling. The tests were repeated three times for each set of samples deposited at 500, 600, 700, and 800 °C.

## 3. Results and Discussion

### 3.1. Microstructural Characteristics

**Figure 1:**
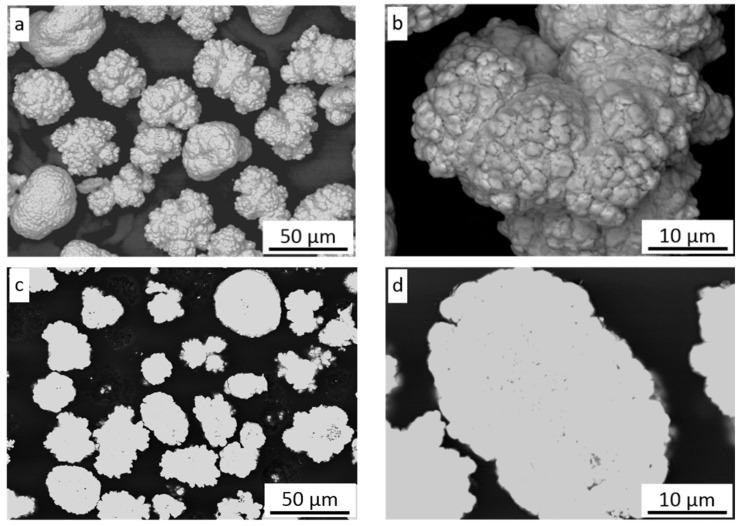
Ni powder: (**a**) morphology of grains, (**b**) surface morphology of grains, (**c**) cross-section of grains, (**d**) high magnification of grain cross-section.

Figure 1a shows the morphology of the nickel powder. Most grains possess an irregular spherical shape with a rugged surface and consist of fine 1–2 µm particles (Figure 1b) as a result of precipitation and aggregation during the hydrogen reduction method [29]. The cross-section of powder grains shows their dense microstructure without any inclusions (Figure 1c); however, some grains reveal small pores inside (Figure 1d).

Sample surfaces were scanned and measured before cold spraying to estimate their influence on the adhesion and mechanical properties of nickel coatings. Their detailed parameters are shown in Table 1.

**Figure 2:**
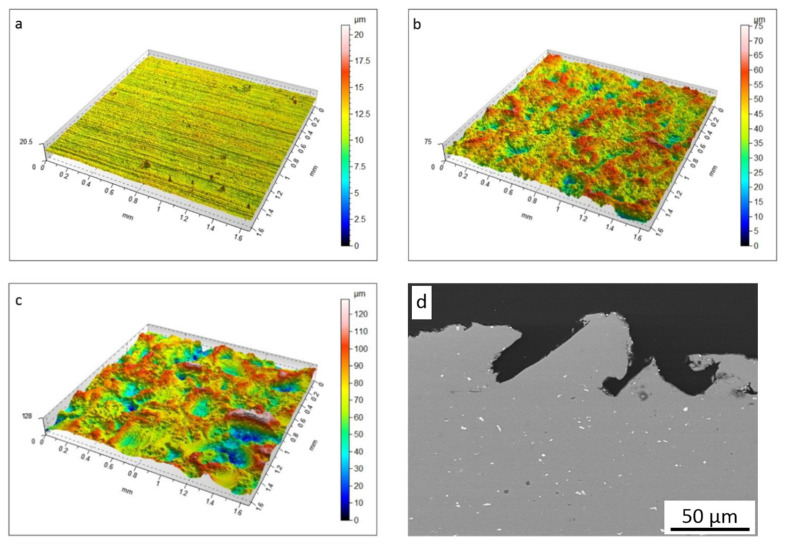
Geometry of Al 7075 substrate surface: (**a**) NG, (**b**) MG, (**c**) CG, (**d**) CG cross-section.

The NG sample represents a typical flat surface (Sa = 0.41 m) of Al 7075 alloy after cold rolling. Subsequent grit-blasting pretreatment caused significant diversity in surface geometry. The highest arithmetic mean of the surface height Sa of the MG surface (Figure 2b) was significantly higher and grew two times for the CG surface (Figure 2c) as a result of large-sized corundum impact. A similar relationship was observed for the mean squared surface height, Sq.

The negative value of asymmetry of the surface, Ssk, indicated that all surfaces were flattened and occurring peaks were rounded. Kurtosis Sku is responsible for steep irregularities and defects. A value of kurtosis over three indicated that the distribution of profile ordinates corresponds to a higher concentration around the mean value, which is clearly visible for the NG coating. The next parameters: the maximum peak Sp, the maximum valley Sv, and the maximum height Sz, significantly increase with the size of the applied corundum grain. The cross-section of the CG sample (Figure 1d) confirmed not only the very high roughness of the surface but also showed deep micropits and hidden microcaves, which were not revealed by the applied white light interferometry system. The horizontal surfaces of all samples were the same (Table 1), but two surfaces became rough after grit-blasting. The area of the MG and CG samples increased by 15% and 30%, respectively. Yin et al. [23] reported that such CG surface geometry makes it difficult to create complete contact with striking particles. As a result, the real oxide-free touch is decreased and does not ensure strong metallurgical bonding.

**Figure 3:**
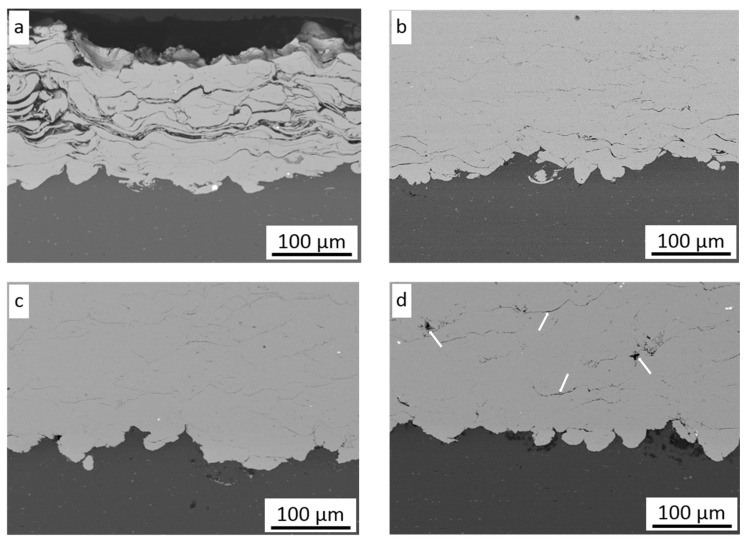
Microstructure of CS Ni coatings sprayed on the CG surface with nitrogen at (**a**) 500 °C, (**b**) 600 °C, (**c**) 700 °C, (**d**) 800 °C, white arrows indicate coating discontinuities.

The deposition of nickel needs a very high velocity and depends on particle size [30]. Bae et al. [31] reported that only powder preheated at 600 °C with a medium size distribution of 37–52 µm allows 95% deposition efficiency to be obtained. The initial parameters for cold spraying Ni coatings were adopted on the basis of conducted experiments with the same cold spray system [32]. The first attempts at 500 °C were not rewarding; however, a nickel coating was formed, but its quality (Figure 3a) was very poor and deposition efficiency very low. Although the first layer showed excellent adhesion to the substrate and intersplat cohesion, the following layers revealed very poor cohesion (Figure 3a) despite grit-blasting with coarse corundum grains. The coating thickness reached a maximum of 0.89 mm for the MG sample, and after that, only the process of the rebound and erosion particles occurred, and the growth process was stopped. The same phenomena were observed not only for the CG sample but also in the case of the two other NG and MG samples. During the cold spray process, the coating is formed as a result of three independent processes that occur simultaneously: grit-blasting, spraying of coating, and shot peening.

Exceeding the critical velocity by powder particles is necessary to deposit the coating. Because the powder for cold spraying consists of different grains and velocity, distribution across the spray stream varies, and not all particles reach critical velocity. The three above-mentioned phenomena in different proportions depend on cold spray parameters and influence the process of coating formation. Proportions between these processes determine the deposition efficiency of the coating. The value of critical velocity for metal particles based on their thermomechanical properties can be calculated from an equation elaborated by Schmidt et al. [33]. Critical velocity is also affected by substrate properties (geometry, oxide film, hardness, etc.) [1,26]. This confirms that the optimal cold spray parameters necessary for the deposition of the first layer may not be the same as those required for the rest of the coating [34]. Papyrin et al. [35] reported a significant effect of preheating gas temperature on the deposition efficiency of nickel. Further increasing preheating gas temperature from 500 to 800 °C causes higher gas velocity and particle impact velocity, resulting in a considerable increase in coating thickness from 0.57 mm to 3.06 mm (Table 2).

**Figure 4:**
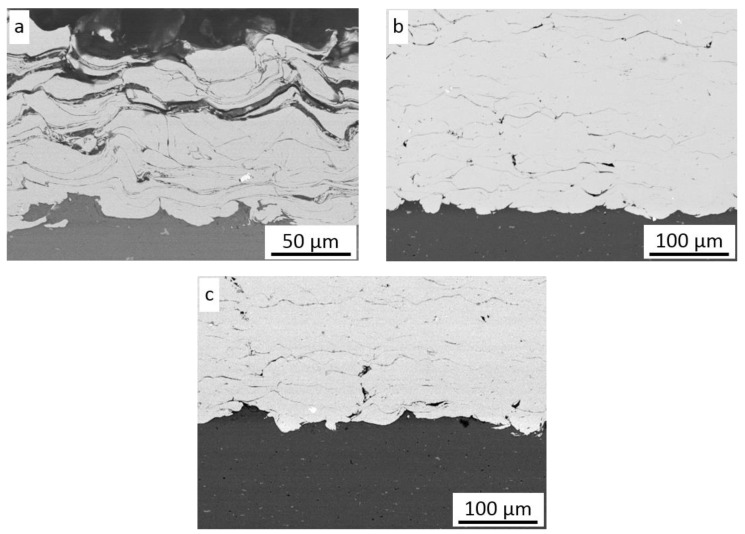
Microstructure of CS Ni coatings sprayed on the surface with nitrogen at (**a**) NG/500 °C, (**b**) NG/600 °C, (**c**) MG/600 °C.

The substrate preparation had a negligible effect on the coating thickness, and in all cases of surface pretreatment, the values were nearly the same. As presented in Figure 3a–d, all particles were severely deformed and showed very good adhesion to the substrate in the whole range of preheating gas temperatures. However, four surfaces were grit-blasted with coarse corundum grain, and rough large surfaces were created, while no voids were detected at the interface between the particle and surface, as reported by Yin et al. [23]. The microstructure of coatings presented in Figure 3b–d shows severely deformed grains despite significant growth of gas preheating temperature and significant differences in deposition efficiency of the sprayed coatings. All deposited particles are highly elongated, and there are no distinct differences in their shape, so it can be assumed that all particles, which exceed critical velocity are subject to deformation in the same way. The higher the preheating temperature (e.g., particle velocity), the wider the range of particles that reach and overcome critical velocity, so coating thickness increases rapidly. Some intersplat cracks and pores (white arrows) were presented across the thickness of coatings, which can indicate reduced cohesion between deformed particles. Such a phenomenon was observed in the top layer and attributed to a small degree of plastic deformation and flattening of the impacted particles [14,31]. Figure 4a presents a higher magnification of the cold-sprayed nickel coating on the substrate in delivery condition. In this case, the surface of the Al 7075 alloy was smooth (Sa = 0.41 µm) and only decreased before spraying Ni coating. The lack of any oxides at the interface confirms that the first particles at the outer diameter of the nozzle possess a velocity lower than the critical velocity, and only the process of grit-blasting by striking particles occurs.

**Figure 5:**
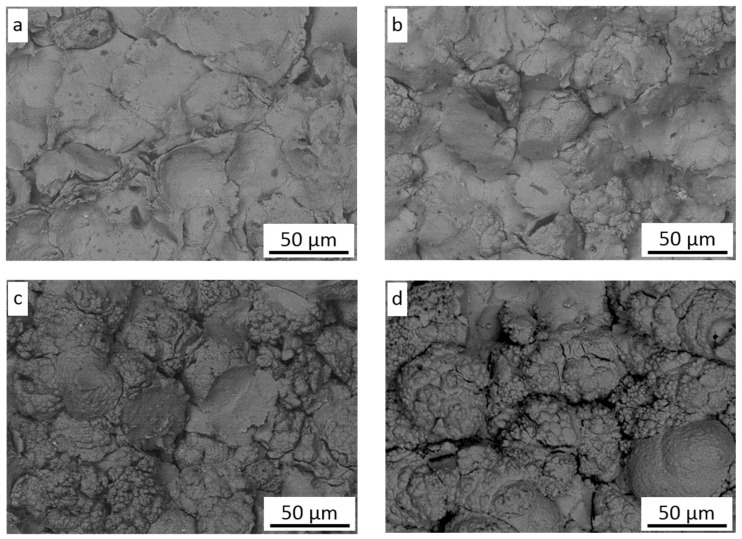
Morphology of CS Ni coating surface sprayed on the NG surface with nitrogen at (**a**) 500 °C, (**b**) 600 °C, (**c**) 700 °C, (**d**) 800 °C.

The lowest particle velocity was enough to remove all contaminations from the surface and activate the substrate to create very good adhesion. There are no voids, pores, or inclusions at the interface, just as in the case of samples prepared by medium and coarse grit-blasting. A comparison of the interface roughness at the same temperature is presented in Figure 3b and Figure 4b,c. Significant differences in substrate geometry before cold spraying (Figure 2) were reduced by the shock impact of nickel particles, which rebound and create craters in both grit-blasted surfaces [23]. The influence of gas preheating temperature, e.g., the velocity of nickel particles during impact, on surfaces in the delivery condition is presented in Figure 5. Surface morphologies showed distinctly different particle behavior. The firmly flattened and weakly bonded splats are clearly visible on the surface of the coating sprayed with the lowest preheated temperature of nitrogen (Figure 5a).

The edges of splats do not adhere to underlying splats, and their cohesion is very poor, which is also confirmed by Figure 3a and Figure 4a. The dominant process is tamping by striking particles, with some splats being additionally concave (white arrows) as a result of this phenomenon. The arithmetic mean of the surface height Sa of this surface (Table 3) is comparable to Sa of coarse grit-blasting, which indicates the similarity of both processes. The increase in temperature causes a limitation of the tamping process; however, weakly-bonded splats are still present on the surface (Figure 5b). Despite the existence of concave splats, the first splats with a rough surface (white arrows) deriving from original nickel grains are clearly visible. The parameter S_a_ = 35.94 µm significantly increases, which implies considerable growth in the surface roughness as a result of the coating thickness rise caused by the augmentation of deposition efficiency. The surface of coatings sprayed with nitrogen preheated to 700 °C consists mainly of well-bonded splats with a rough surface (Figure 5c) and only single traces of reflected particles are present (white arrow). The arithmetic mean of the surface height (45.64 µm) increased as a result of the clearly visible rough deformed grains. The highest temperature caused a significant growth in particle velocity and the creation of highly-flattened particles (Figure 5d). Some fractured splats were present (white arrows), which may have resulted from dynamic compressive and shear failure [31]. The parameter S_a_ = 31.71 µm significantly decreased as a result of strong particle deformation.

**Figure 6:**
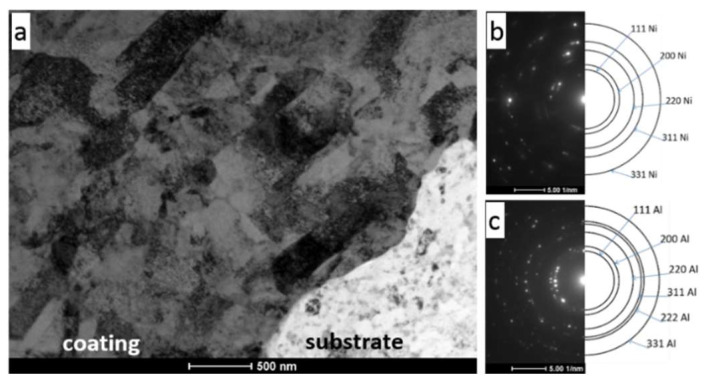
(**a**) BF TEM image of part of the Ni coating/7075 Al alloy substrate system, (**b**) SAD pattern corresponds to the Ni coating, (**c**) SAD pattern corresponds to the aluminium alloy substrate.

**Figure 7:**
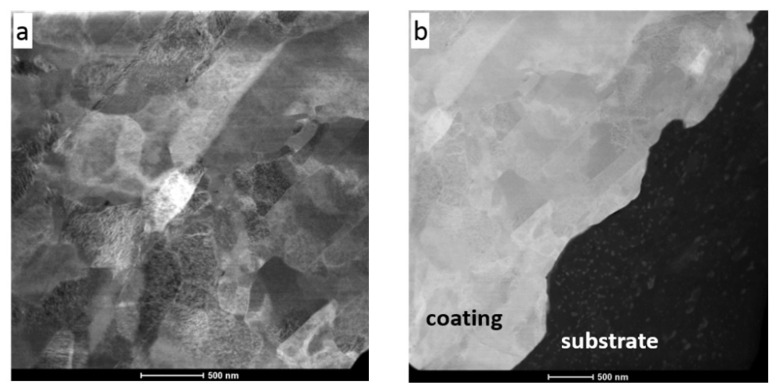
STEM images of (**a**) Ni coating, (**b**) area showing coating/substrate interface.

**Figure 8:**
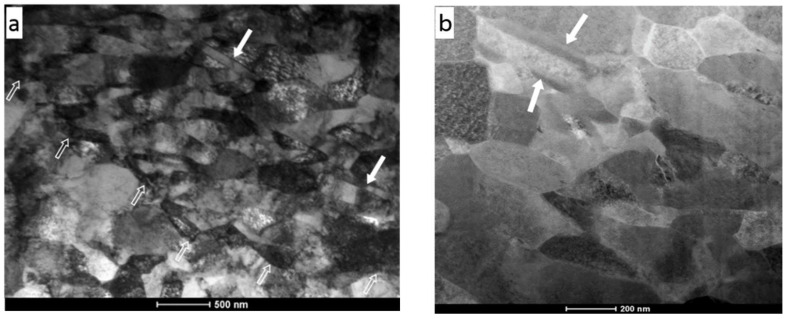
(**a**) BF TEM image and (**b**) STEM image of the sample cut out from the region lying in the upper part of the Ni coating—near the coating surface. The white-filled arrows indicate twin grains, while the white hollow arrows show a boundary of Ni splat.

Investigations of microstructure using TEM were performed in two regions of the CS Ni coating with nitrogen preheated at the temperature of 700 °C: one covering an area of the interface of the coating and 7075 Al alloy substrate (Figure 6 and Figure 7) and the other localized near the surface of the Ni coating (Figure 8). This enabled both an investigation of the coating microstructure and an observation of its adhesion to the substrate. 

The coating revealed a splat (layer) structure, which was strictly connected with the specifics of deposition during cold spraying. The splats were substantially parallel to the substrate, which is clearly visible in Figure 6 (bright field (BF) TEM image) and Figure 7 (scanning transmission electron microscopy (STEM) image). Ni grains with different shapes (elongated, equiaxed) and sizes of a few dozen to several hundred nanometers were present in the splats. Highly distorted and deformed parts of splats were observed near the particle/particle interface region due to the high-velocity impact of deposited particles. Ni grains in the vicinity of this interface region were slightly refined relative to the grains in the inner region of the deposited particles. In this way, a meaningful fragmentation of the original grain microstructure of Ni powder used as the feedstock (Figure 1) was observed.

The bonding of particles to the substrate during cold spraying can be attributed to the extremely high strain rate of plastic deformation at the particle/substrate interface [36]. Additional input into the substrate deformation process is also derived from its blasting by large-sized Al_2_O_3_ sand prior to cold spraying. The presence of very small grains in the substrate structure near the coating/substrate interface (Figure 6a) is evidence of this phenomenon. Additionally, the selected area diffraction (SAD) pattern of the substrate (Figure 6c) showed reflections lying along the rings corresponding to small individual grains of the Al phase.

The coating revealed a very dense microstructure, which indicated that the impacting powder particles formed a good bond with the previously deposited layer. The white hollow arrows visible in Figure 8 show the boundary of Ni splats. It is worth emphasizing that nanotwins (white-filled arrows) formed by deformation twinning were also observed in the coating microstructure. This is in accordance with the previous examinations reported by Bae [37].

**Figure 9:**
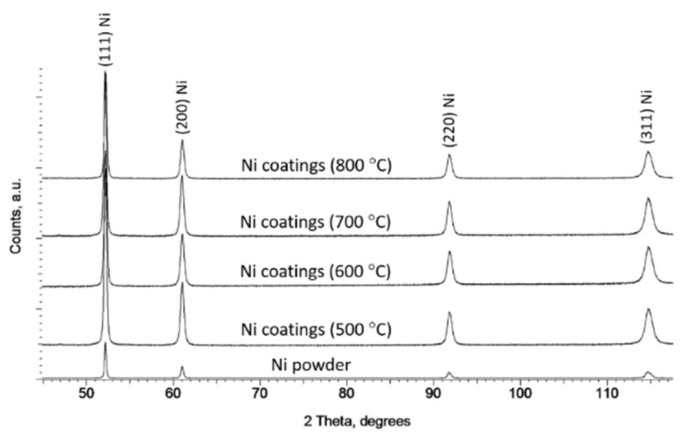
X-ray diffraction patterns obtained for both the Ni powder feedstock and coatings cold sprayed at various nitrogen temperatures: 500 °C, 600 °C, 700 °C, 800 °C.

Figure 9 presents the X-ray diffraction patterns obtained for both the Ni powder feedstock and coatings cold sprayed on NG surfaces at several preheated nitrogen temperatures (500, 600, 700, and 800 °C). The same patterns were carried out for Ni coatings sprayed on the MG and CG Al 7075 substrates. As expected, all patterns showed no distinct shift or broadening of plane reflections. A relatively low peak intensity obtained for the powder resulted from the use of different diffractometers and measurement parameters compared to the coatings.

We calculated the lattice parameters based on the measurements, which are presented in Table 4.

### 3.2. Mechanical Characteristics

**Figure 10:**
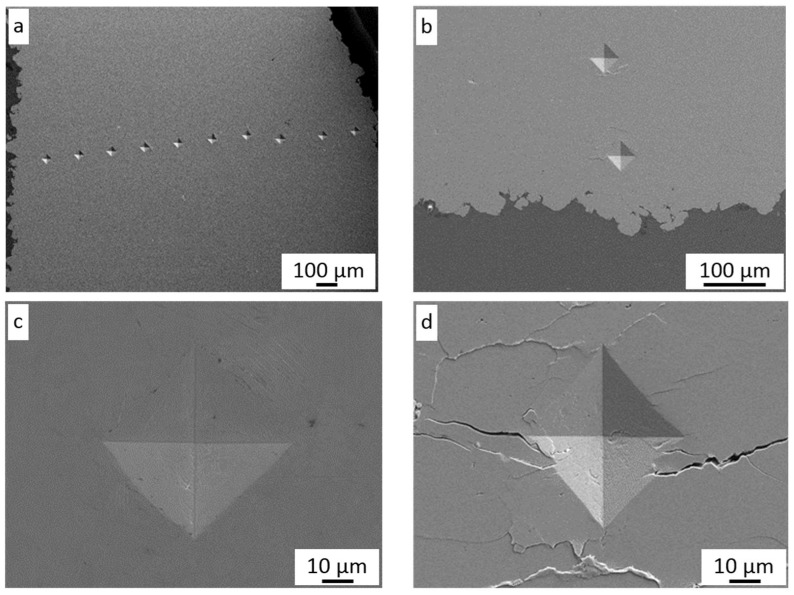
HV microhardness imprints: (**a**) across the CS Ni coating (CG/600 °C), (**b**) inter-plat cracks on the CS Ni coating near to the Al 7075 substrate, (**c**) Ni bulk material, (**d**) high magnification of inter-splat cracks in the middle of the CS Ni coating.

The microhardness measurements were carried out on polished cross-sections of coatings along their thickness (Figure 10a). As can be seen in Figure 10b, around each imprint, there are visible microcracks (white arrows). These microcracks are created along splat boundaries under an external load put on the indenter, which is clearly visible under higher magnifications in Figure 10d. This suggested weak intersplat bonding and high levels of residual stress in the coatings. Such a phenomenon was caused by tamping by nickel particles with the largest dimensions, which did not reach critical velocity [23,31].

The very high roughness of Ni coatings (Table 3) with visible craters confirmed this assumption. On the other hand, the application of finer powders allowed the microstructure to be improved [38,39]. Moreover, quiet crackling was heard during indentation, which confirmed very high stress inside the coatings caused by particles impacting the previously deposited layer with very high velocity. Such a phenomenon did not appear on the surface of bulk nickel where the plastic flow of material (white arrow) was seen around the imprint (Figure 10c). Residual stresses promoted crack initiations and propagation, leading to a significant reduction in the component life.

**Figure 11:**
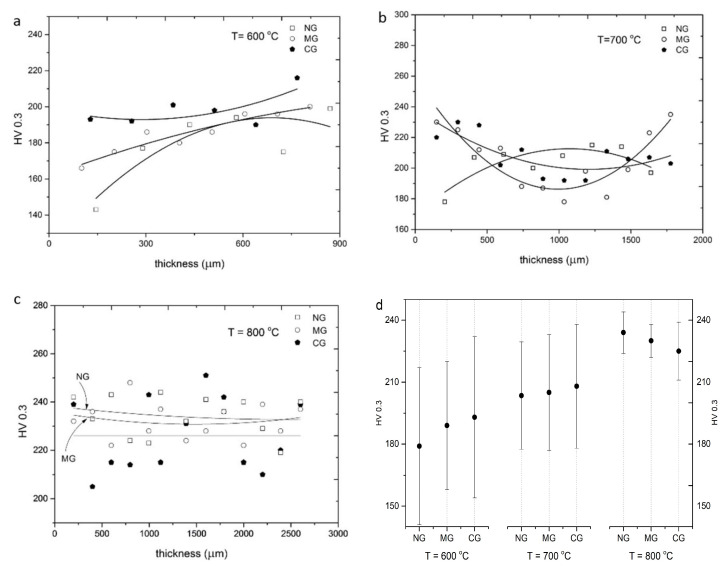
Microhardness of CS Ni coatings with nitrogen gas at: (**a**) 600 °C, (**b**) 700 °C, (**c**) 800 °C, (**d**) collected results.

The change in coating microhardness along with thickness is shown in Figure 11a–c. The microhardness values did not reveal any dependencies on indentation places near the substrate or at the top of the deposit. All hardness measurement results are collected in Figure 11d. The surface condition of Al 7075 samples before cold spraying does not influence the level of coating microhardness. However, microhardness slightly increases with growth in surface roughness in the case of 600 and 700 °C preheated gas, while it decreases at 800 °C. Very high standard deviations confirmed significant differences in the mechanical properties of cold-sprayed nickel coatings directly related to large amounts of cracks in coatings. The highest value of 234 HV 0.3 reached the coating sprayed at 800 °C on the NG sample. Bae et al. [31] reported microhardness in the range of 218–313 depending on the cold spray parameters and ranges of powder grains. This maximum value is distinctly higher than 211 HV obtained by [40] and 233 HV and 238 HV reported by [41]. All values are significantly higher than the microhardness of bulk nickel of 128 HV0.3, which is attributed to strain hardening of cold-sprayed nickel coatings.

**Figure 12:**
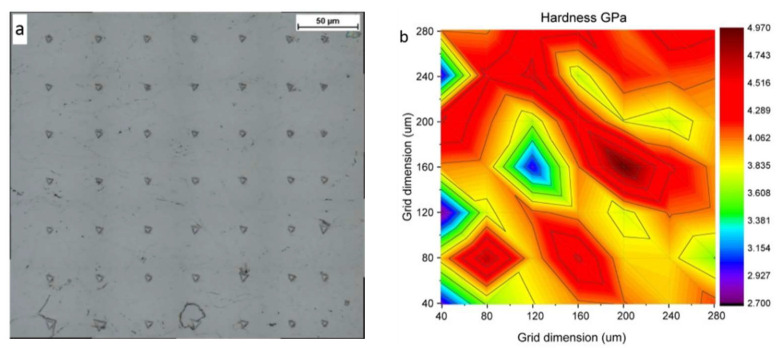
Cross-section of CS Ni coatings: (**a**) square array of nanoindentations, (**b**) a contour plot of nanohardness versus position within the coating.

**Figure 13:**
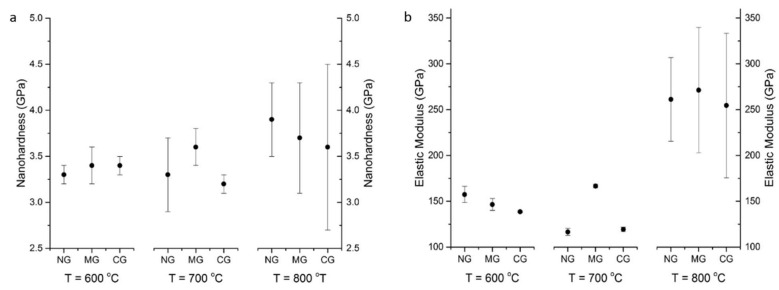
CS Ni coatings: (**a**) nanohardness, (**b**) elastic modulus.

In order to examine the influence of different local deformation of nickel grains on the mechanical properties of cold-sprayed coatings, nanoindentation was carried out. An array of 7 × 7 indentations was carried out on a polished surface with an indent spacing of 40 μm in the middle of each coating (Figure 12a). The Oliver and Pharr method was applied to calculate hardness and elastic modulus on the base of the load vs. depth curves [42]. A nanohardness contour map presents changes in the investigated area of 240 × 240 μm (Figure 12b). Such maps were created for each coating, and the mean values of nanohardness and elastic modulus are presented in Figure 13a,b, respectively.

The mean hardness of CG coatings for temperatures 600, 700, and 800 °C was 197.6 ± 6.7, 208.4 ± 13.2 and 225.3 ± 14.4, respectively.

Contour maps of nanohardness and elastic modulus distinctly showed significant differences in the regions exhibiting higher and lower values of the investigated parameters. Tested areas were free of voids and porosity, and the obtained results are of different local deformation of nickel grains. The indentations cover the surface of a few dozen splats and showed values obtained in the interior of splats with different degrees of deformation as well as particle/particle interfaces. The obtained nanohardness range does not depend on the surface condition before cold spraying. A light increase in nanohardness can be observed according to the growth in preheating temperature of nitrogen from 3.3 GPa for the NG sample at 600 °C to 3.9 GPa for NG at 800 °C. This value is higher than 3.4 GPa reported by [33] but distinctly lower than 9.7 obtained by Aldenstein et al. for nanocrystalline nickel powder [14]. The nanohardness of bulk nickel of 2.3 GPa is significantly lower, which is attributed to the cold working process, as in the case of microhardness. A clear increase in elastic modulus is present at 800 °C. The higher velocity of nickel particles caused a high degree of deformation and anisotropic microstructure with a high value of standard deviations. 

**Figure 14:**
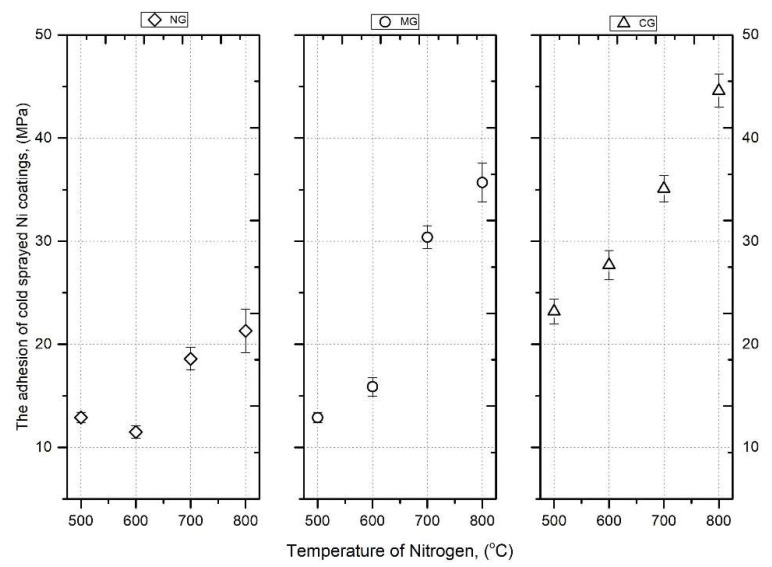
The adhesion of cold-sprayed Ni coatings vs. nitrogen.

Figure 14 presents the obtained results of coating adhesion as a function of gas temperature and the type of substrate preparation. All samples showed adhesion failures, i.e., the coating separated from the substrate. The investigated adhesion to non-blasted coatings (NG) exhibited only slight variations with respect to the temperature of the working gas preheating. At the temperatures of 500 and 600 °C, the adhesion remained practically constant. This could be attributed to the process of grain collisions between Ni particles and the hard Al 7075 substrate, which has an elastic modulus of 155.0 ± 9.0 GPa. Increasing the temperature to 700 °C elevated the kinetic energy of the particles, resulting in an enhancement in the coating adhesion, reaching a value of 18.6 ± 1.1 MPa. At a temperature of 800 °C, this adhesion further increased to 21.3 ± 2.1 MPa.

The adhesion of the deposited Ni coatings on the medium grit-blasted substrate (MG) at temperatures of 500 and 600 °C was comparable to the adhesion of the non-blasted coating (NG). However, increasing the working gas temperature to 700 and 800 °C significantly affected the adhesion value, which increased from 16 to 35.7 ± 1.9 MPa.

In the case of the Al 7075 substrate (CG), the adhesion value at lower temperatures remained at the level of the adhesion forces of the MG coating. However, at a temperature of 800 °C, the adhesion significantly increased, reaching a value of 44.6 MPa. This change can be explained by the increase in kinetic energy of the Ni powder grains. The crucial factor is the larger size of Ni grains, which results in greater mass and, consequently, higher momentum. This increased momentum is responsible for the plastic deformation of both the grains and substrate. Coarse grid-blasting of the substrate (CG) leads to an increase in the actual contact surface area between the coating and substrate. The high energy of the impacting nickel grains could lead to a reduction in the distance between Ni atoms and the substrate, thereby potentially increasing the electrostatic (electrodynamic) interaction and enhancing adhesion forces [16,43].

## 4. Conclusions

Nickel coatings were successfully sprayed on the Al 7075 substrate with three different levels of surface roughness. Moreover, the preheating temperature of nitrogen was increased from 500 to 800 °C. The SEM analysis of coatings revealed a dense microstructure, showing very good adhesion of the impacting powder particles and good bonding between the deposited layers. Weaker intersplat bonding was caused by tamping by nickel particles with the largest dimensions. Results show that the surface preparation had a negligible effect on coating adhesion and microstructure. The initial significant differences in surface roughness of substrates were diminished by grit-blasting with the first striking nickel particles; however, the increased area of the sample surface after coarse grit-blasting could be beneficial for coating adhesion. The TEM observations revealed Ni grains with different shapes (elongated, equiaxed) and sizes of a few dozen to several hundred nanometers present in the splats. Nanotwins formed by deformation twinning were also observed in the coating microstructure. The results show that the surface preparation had a significant effect on coating adhesion and microstructure. On the other hand, there were no distinct dependencies of surface roughness on the thickness of the cold-sprayed nickel coating. An increase in temperature caused significant growth in coating thickness as a result of the powder grains’ higher velocity. Moreover, higher gas temperature resulted in enhancement of micro- and nanohardness and elastic modulus. The adhesive bond strength of Ni coatings in the tested temperature range increased with the increase in the surface roughness of the substrate. For the Al 7075 coarse grit-blasted (CG) substrate, the adhesion reached the highest value of 44.6 MPa when the working gas was at a temperature of 800 °C.

## Figures and Tables

**Table 1:** Geometry of Al 7075 surface parameters.

Parameter	Sample Surface Treatment		
	NG	MG	CG
S_a_	0.41	6.20	14.67
S_q_	0.55	8.05	18.76
S_sk_	−1.50	−0.77	−0.44
S_ku_	25.27	4.26	3.37
S_p_	8.80	30.57	56.41
S_v_	12.09	44.78	72.37
S_z_	20.90	75.35	128.78
Horizontal surface, mm^2^	2.76	2.76	2.76
Developed surface, mm^2^	2.78	3.20	3.62
Depth, m	12.18	50.93	94.30
Volume, mm^3^	0.0008	0.0106	0.0259

**Table 2:** Thickness of CS Ni coatings.

Surface Treatment/ Gas Temp	Coating Thickness, mm		
	NG	MG	CG
Ni coating, 500 °C	0.11 ± 0.01	0.15 ± 0.02	0.11 ± 0.02
Ni coating, 600 °C	1.32 ± 0.02	1.34 ± 0.02	1.33 ± 0.02
Ni coating, 700 °C	2.28 ± 0.05	2.19 ± 0.05	2.11 ± 0.03
Ni coating, 800 °C	3.11 ± 0.05	3.11 ± 0.05	3.14 ± 0.05

**Table 3:** Surface roughnes of CS Ni coatings.

Surface Treatment/ Gas Temp	Surface Roughness Sa, m		
	NG	MG	CG
Ni coating, 500 °C	12.44	13.67	14.53
Ni coating, 600 °C	35.94	41.91	101.05
Ni coating, 700 °C	45.64	42.95	42.62
Ni coating, 800 °C	31.71	41.71	28.68

**Table 4:** Lattice parameter of the tested Ni coatings.

Surface Treatment/ Gas Temp	Lattice Parameter, Å		
	NG	MG	CG
Ni coating, 500 °C	3.5245 ± 0.0004	3.5226 ± 0.0004	3.5244 ± 0.0004
Ni coating, 600 °C	3.5241 ± 0.0004	3.5235 ± 0.0004	3.5256 ± 0.0004
Ni coating, 700 °C	3.5229 ± 0.0004	3.5244 ± 0.0004	3.5247 ± 0.0004
Ni coating, 800 °C	3.5239 ± 0.0004	3.5225 ± 0.0004	3.5226 ± 0.0004
Powder	3.5247 ± 0.0004		

## Data Availability

Not applicable.
